# Spin accumulation assisted by the Aharonov-Bohm-Fano effect of quantum dot structures

**DOI:** 10.1186/1556-276X-7-510

**Published:** 2012-09-17

**Authors:** Wei-Jiang Gong, Yu Han, Guo-Zhu Wei, An Du

**Affiliations:** 1 College of Sciences, Northeastern University, Shenyang, 110819, China; 2 Department of Physics, Liaoning University, Shenyang, 110036, China; 3 International Centre for Materials Physics, Chinese Academy of Sciences, Shenyang, 110016, China

**Keywords:** Spin accumulations, Aharonov-Bohm-Fano effect, quantum dot, Coulomb interaction, 73.63.Kv, 71.70.Ej, 72.25.-b

## Abstract

We investigate the spin accumulations of Aharonov-Bohm interferometers with embedded quantum dots by considering spin bias in the leads. It is found that regardless of the interferometer configurations, the spin accumulations are closely determined by their quantum interference features. This is mainly manifested in the dependence of spin accumulations on the threaded magnetic flux and the nonresonant transmission process. Namely, the Aharonov-Bohm-Fano effect is a necessary condition to achieve the spin accumulation in the quantum dot of the resonant channel. Further analysis showed that in the double-dot interferometer, the spin accumulation can be detailedly manipulated. The spin accumulation properties of such structures offer a new scheme of spin manipulation. When the intradot Coulomb interactions are taken into account, we find that the electron interactions are advantageous to the spin accumulation in the resonant channel.

## Background

Quantum dot (QD), especially coupled-QD system (i.e., the QD molecule), is of fundamental interest in physics and possesses potential applications, such as quantum logic gates[1,2]. As a result, many experimental and theoretical works have paid so much attention to the electron transport properties of various multi-QD systems in the past decades [3-10]. Besides, the progress of nanotechnology enables researchers to fabricate a variety of coupled-QD structures with sizes smaller than the electron coherence length [11]. This also accelerates the development of researches on the coupled-QD characteristics.

With respect to the coupled-QD structures, the typical one is Aharonov-Bohm (AB) interferometer with one QD or whose individual arm is of one QD, respectively [12-38]. In such kind of structure, the AB phase can adjust the quantum interference, leading to abundant interesting results. Kobayashi et al. performed significant work to study the quantum interferences in the AB interferometers with embedded QDs [18-20]. According to their conclusions, the Fano effect, which manifests itself in the asymmetric lineshape of the transport spectrum, can be observed in such structures by constructing nonresonant and resonant channels for electron transmission. Moreover, they showed that the orientation of the Fano lineshape changes periodically with the magnetic flux. Due to this reason, in the AB interferometer with QDs, the AB-Fano interference attracted more attention and was further investigated [22,23]. On the other hand, lots of theoretical investigations about electron transport behaviors of the AB interferometer have been reported. It was found that the interplay between the AB-Fano effect and the other mechanisms, e.g., Kondo physics and the spin-orbit interaction, indeed causes many interesting phenomena [24-38].

Electron not only has a charge but also spins with $s=\frac{1}{2}$; accordingly, the electron spin in the QD has been suggested as an ideal candidate for the qubit. Then, the coherent generation and control of electron spins in QDs has recently become one main subject in spintronics [39-42]. Various schemes have been proposed: by considering the spin transport and spin accumulation in QDs, based on the magnetic means, the spin-obit interaction, etc.[43-49]. Since in QD structures, the quantum interference contributes significantly to the electron motion properties, it is natural to question about the role of quantum interference on the spin accumulation. However, to our knowledge, little attention has been paid to such an issue so far. In this work, we choose the AB interferometer with embedded QDs and clarify the effect of a typical interference manner, i.e., the AB interference, on the spin manipulation in QDs. In doing so, we introduce a symmetric spin battery to the interferometer by considering the chemical potentials of the leads to be $\mu_{\mathrm{L\sigma}}=\epsilon_{F}+\sigma \frac{eV_{s}}{2}$ and $\mu_{\mathrm{R\sigma}}=\epsilon_{F}+\bar{\sigma }\frac{eV_{s}}{2}$[50-54]. We intend to investigate the role of quantum interference in adjusting the spin-bias-induced spin accumulation. *ϵ*_*F*_ is the Fermi level of the system at the zero-spin-bias case, and *V*_*s*_is the magnitude of the spin bias. Due to the progress in experiment, such a scheme can be realized by injecting the charge current from a ferromagnetic source ( or a magnetic field ) into the leads of the QD structure [55-60]. Consequently, we find that to achieve the spin manipulation in the QDs of the AB interferometer, a finite magnetic flux and a nonresonant channel are prerequisites. Namely, the AB-Fano interference, not only the AB effect, is a necessary condition to realize the spin accumulation in the QDs. Also, the spin accumulation can be adjusted by varying the quantum interference of the interferometer. Therefore, we believe that such a structure is a promising candidate for spin manipulation.

## Model and numerical results

The Hamiltonian that describes the electron motion in the AB interferometer can be written as

(1) $$H=H_{L}+H_{R}+H_{D}+H_{T}.$$

*H*_*α*_(*α*=*L*,*R*) is the Hamiltonian in lead-*α*. *H*_*D*_ is the Hamiltonian in the QDs, and the last term, *H*_*T*_, denotes electron traveling between the two leads. *H*_*α*_takes a form as $\left(H_{\alpha }=\underset{\mathrm{k\sigma}}{\sum }\epsilon_{\mathrm{\alpha k\sigma}}c_{\mathrm{\alpha k\sigma}}^{‡}c_{\mathrm{\alpha k\sigma}}\right)$, where $\left(c_{\mathrm{\alpha k\sigma}}^{‡}(c_{\mathrm{\alpha k\sigma}})\right)$ is the creation (annihilation) operator corresponding to the basis in lead-*α*. *ϵ*_*αkσ*_ is the single-particle level. Since we investigate the electron properties of two AB interferometers with one QD or two QDs, the expressions of *H*_*D*_and *H*_*T*_ will be determined by the geometries of the interferometers.

### The one-QD AB interferometer

**Figure 1:**
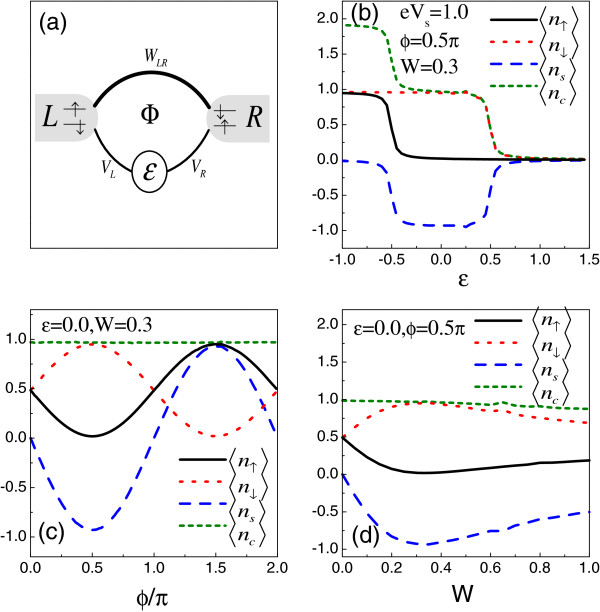
**The AB interferometer of one QD. (a)** Schematic of an AB interferometer with an embedded QD. **(b, c, d)** The average electron occupation number and spin accumulation in QD affected by the structure parameters. The relevant parameters are taken to be *ρ* = 1, |*V*_*α*_|= 0.1, and *eV*_*s*_ = 1.0.

We first focus on the AB interferometer of one QD, whose schematic is shown in Figure 1a. Then in such a case, $\left(H_{D}=\underset{\sigma }{\sum }\epsilon d_{\sigma }^{‡}d_{\sigma }+Un_{↑}n_{↓}\right)$ and $\left(H_{T}=\underset{\mathrm{k\sigma}}{\sum }\;W_{\mathrm{LR}}c_{\mathrm{Lk\sigma}}^{‡}c_{\mathrm{Rk\sigma}}+\underset{\mathrm{\alpha k\sigma}}{\sum }V_{\alpha }c_{\mathrm{\alpha k\sigma}}^{‡}d_{\sigma }+\text{h.c.}.d_{\sigma }^{‡}(d_{\sigma })\right)$ are the creation (annihilation) operator of electron in the QD, and *ϵ*is the energy level of QD. *U* is the intradot electron interaction strength. *W*_*LR*_ denotes the direct transmission between the leads, and *V*_*α*_ represents the coupling between the QD and lead-*α*.

The electron properties can be evaluated by using the nonequilibrium Green function technique. In the Green function space, the average electron occupation number of the QD is denoted as [61,62]

(2) $$\langle n_{\sigma }\rangle =-\frac{i}{2\Pi }\int \mathrm{d\omega}G_{\mathrm{dd},\sigma }^{<}(\omega ).$$

*G*^<^ is the lesser Green function, which can be obtained from the Dyson equation

(3) $$G^{<}(\omega )=(1+\Sigma^{r}G^{r})g^{<}(1+\Sigma^{a}G^{a})+G^{r}\Sigma^{<}G^{a}.$$

*G*^*r*^ and *G*^*a*^ are the retarded and advanced Green functions, respectively. Due to the presence of electron interaction, the Green function is difficult to solve. However, if the system temperature is higher than the Kondo temperature, the electron interaction term can be included by using the Hubbard-I approximation [61-63]. In this work, we would like to consider the case of weak electron correlation; then, the retarded Green function can be analytically solved within the Hubbard-I approximation, i.e.,

(4) $${\left[G_{\sigma }^{r}\right]}^{-1}=\left[\begin{array}{ccc} \;g_{L}^{r-1} & -W_{\mathrm{LR}} & -V_{L}\\ -W_{\mathrm{LR}}^{*} & g_{R}^{r-1} & -V_{R}\\ -V_{L}^{*} & -V_{R}^{*} & \;g_{\mathrm{d\sigma}}^{r-1} \end{array},\;\right]\;,$$

where $\left(g_{\alpha }^{r}\right)$ is the Green function of the isolated lead-*α*. Due to the continuum states in the leads, we write $\left(g_{\alpha }^{r}=-\mathrm{i\Pi \rho}(\omega )\right)$ with *ρ*(*ω*) being the density of states of the leads. $\left(g_{\mathrm{d\sigma}}^{r}\right)$, the Green function of the isolated QD, can be written as $g_{\mathrm{d\sigma}}^{r}=\frac{1-\langle n_{\bar{\sigma }}\rangle }{\omega -\epsilon +i0^{+}}+\frac{\langle n_{\bar{\sigma }}\rangle }{\omega -\epsilon -U+i0^{+}}$ in the Hubbard-I approximation. In this structure, $\left(G_{\mathrm{dd},\sigma }^{<}\right)$ can be expressed as $\left(G_{\mathrm{dd},\sigma }^{<}=\underset{\alpha }{\sum }G_{\mathrm{d\alpha},\sigma }^{r}g_{\alpha }^{r-1}g_{\alpha \sigma }^{<}g_{\alpha }^{a-1}G_{\mathrm{\alpha d},\sigma }^{a}\right)$, where $\left(g_{\alpha \sigma }^{<}=2\mathrm{i\Pi \rho}(\omega )f_{\alpha \sigma }(\omega )\right)$ is the lesser Green function of the isolated lead-*α* with the Fermi distribution function $\left(f_{\alpha \sigma }(\omega )={\left[\exp\frac{\omega -\mu_{\alpha \sigma }}{k_{B}T}+1\right]}^{-1}\right)$. When the bandwidth of the leads is large enough, the density of states can be viewed as a constant. Accordingly, we have $G_{\mathrm{dd},\sigma }^{<}=\frac{2i}{\Pi \rho }\underset{\alpha }{\sum }|G_{\mathrm{d\alpha},\sigma }^{r}{|}^{2}f_{\alpha \sigma }(\omega )$. As a result, 〈*n*_*σ*_〉 can be re-expressed as

(5) $$\langle n_{\sigma }\rangle =\frac{1}{\Pi^{2}\rho }\underset{\alpha }{\sum }\int \mathrm{d\omega}|G_{\mathrm{d\alpha},\sigma }^{r}(\omega ){|}^{2}f_{\alpha \sigma }(\omega ).$$

Furthermore, by defining $\langle n_{c}\rangle =\underset{\sigma }{\sum }\langle n_{\sigma }\rangle$ and 〈*n*_*s*_〉=〈*n*_*↑*_〉−〈*n*_*↓*_〉, we can investigate the features of the charge and spin in the QD.

With the help of Equation 5, we investigate the average electron occupation number influenced by the structure parameters in Figure 1b,c,d. The system temperature is fixed at *k*_*B*_*T*=0.1. For the other parameters, we choose the spin bias *eV*_*s*_=1.0 and the QD-lead coupling strength |*V*_*α*_|=0.1. In Figure 1b, it is observed that at the case of *ϕ*=0.5*Π*, a spin-up electron can enter the QD only when the QD level decreases to the position of *ϵ*=−0.5. Instead, the QD is able to confine a spin-down electron if *ϵ*<0.5. By comparing the properties of opposite-spin electrons, we might as well consider that the spin-up and spin-down electrons are both in equilibrium, but they ‘feel’ the different ‘Fermi levels’, with the distance between them being the spin bias magnitude. Therefore, in such a structure, the striking spin accumulation can be realized in the QD. Next, in Figure 1c,d, by assuming *ϵ*=0, we present the influence of *ϕ*and *W * on the average occupation number of electron in the QD, respectively. It is observed that with the change of magnetic flux, the average occupation of different-spin electrons show opposite variation features. Different from the result of *ϕ*=0.5*Π*, when the magnetic flux is increased to *ϕ*=1.5*Π*, the QD confines a spin-up electron. Then, the spin accumulation in the QD can be completely adjusted. Alternatively, with *W * increased to *W*=0.3, the spin accumulation proportionally enhances; however, the further increase of *W * will lead to the suppression of spin accumulation.

Since the structure is relatively simple, we try to clarify the numerical result in an analytical way. Accordingly, we write out the expression of $\left(G_{\mathrm{dL},\sigma }^{r}\right)$ by using Equation 4, i.e., $\left(G_{\mathrm{dL},\sigma }^{r}(\omega )=\frac{1}{D_{t}}[g_{L}^{r}W_{\mathrm{LR}}g_{R}^{r}V_{R}+g_{L}^{r}V_{L}]\right)$ and $G_{\mathrm{dR},\sigma }^{r}\;(\omega )=\frac{1}{D_{t}}\;[g_{R}^{r}\;W_{\mathrm{LR}}^{*}\;g_{L}^{r}\;V_{L}\;+\;g_{R}^{r}\;V_{R}]$ with $\left(D_{t}\;=\;g_{L}^{r}\;g_{R}^{r}\;\text{det}\{{\left[G_{\sigma }^{r}\right]}^{-1}\}\right)$. When a local magnetic flux is applied, its effect on the quantum interference can be well defined by writing *W*_*LR*_=*We*^*iϕ*^. Here, *W * is the strength of the lead-lead coupling, and the phase factor $\phi =\frac{\Phi }{\phi_{0}}$ where *Φ* is the magnetic flux with the magnetic flux quantum $\phi_{0}=\frac{\mathrm{hc}}{e}$. In the case of weak QD-lead coupling, e.g., |*V*_*α*_|=0.1, the analytical form of 〈*n*_*σ*_〉 can be approximated as $\langle n_{\sigma }\rangle =\Delta_{-\phi }\frac{\Gamma^{L}}{\Gamma^{L}+\Gamma^{R}}f_{\mathrm{L\sigma}}(\tilde{\epsilon })+\Delta_{\phi }\frac{\Gamma^{R}}{\Gamma^{L}+\Gamma^{R}}f_{\mathrm{R\sigma}}(\tilde{\epsilon })$. *χ*=*ΠρW*, $\left(\tilde{\epsilon }=\epsilon -2\chi \sqrt{{\tilde{\Gamma }}^{L}{\tilde{\Gamma }}^{R}}\cos\phi \right)$, $\Delta_{\pm \phi }=\frac{1+\chi^{2}\pm 2\chi \sin\phi }{1+\chi^{2}}$, and ${\tilde{\Gamma }}^{\alpha }=\frac{\Gamma^{\alpha }}{1+\chi^{2}}$ with $\left(\Gamma^{\alpha }=\Pi |V_{\alpha }{|}^{2}\rho \right)$. The expression of 〈*n*_*c*_〉 and 〈*n*_*s*_〉 can then be obtained, i.e., $\left(\langle n_{c}\rangle =f_{\mathrm{\alpha ↑}}(\tilde{\epsilon })+f_{\mathrm{\alpha ↓}}(\tilde{\epsilon })\right)$ and $\langle n_{s}\rangle =\Delta_{-\phi }\frac{\Gamma^{L}}{\Gamma^{L}+\Gamma^{R}}[f_{\mathrm{L↑}}(\tilde{\epsilon })-f_{\mathrm{L↓}}(\tilde{\epsilon })]+\Delta_{\phi }\frac{\Gamma^{R}}{\Gamma^{L}+\Gamma^{R}}[f_{\mathrm{R↑}}(\tilde{\epsilon })-f_{\mathrm{R↓}}(\tilde{\epsilon })]$. We really find that the average charge occupation in the QD is independent of the presence of spin bias. However, in the case of finite spin bias, the factor *Δ*_±*ϕ*_, which is contributed by the local magnetic flux and direct lead-lead coupling, can adjust the value of 〈*n*_*s*_〉. Also, the spin accumulation is an odd function of *ϕ*, so that the magnitude and sign of spin accumulation can be detailedly adjusted by the change of magnetic flux. Furthermore, we see that when *ΠρW*=±sin*ϕ*=1, the expressions of 〈*n*_*dσ*_〉 and 〈*n*_*s*_〉 can be simplified. For the example of *ΠρW*=1 and $\phi =\frac{\Pi }{2}$, $\langle n_{\sigma }\rangle =\frac{2\Gamma^{R}}{\Gamma^{L}+\Gamma^{R}}f_{\mathrm{R\sigma}}(\epsilon )=f_{\mathrm{R\sigma}}(\epsilon )$. Then, in such a case, the ‘Fermi level’ of the spin-*σ* electron is at the point of *ϵ*=*μ*_*Rσ*_, leading to the result that 〈*n*_*s*_〉=*f*_*R↑*_(*ϵ*)−*f*_*R↓*_(*ϵ*). Next, when the magnetic flux is raised to $\phi =\frac{3\Pi }{2}$, there will be 〈*n*_*dσ*_〉=*f*_*Lσ*_(*ϵ*) and 〈*n*_*s*_〉=*f*_*L↑*_(*ϵ*)−*f*_*L↓*_(*ϵ*). The property of the spin polarization is completely opposite to the case of $\phi =\frac{\Pi }{2}$. Based on such analysis, the spin accumulation in the QD is well understood.

The underlying physics being responsible for the above results is quantum interference. It is known that the interference in the QD ring structure is rather complicated. However, in such a structure, the quantum interference that affects the spin accumulation just occurs between two Feynman paths. This is because $\left(G_{\mathrm{d\alpha},\sigma }^{r}=\tau_{\mathrm{d\alpha},\sigma }^{\left(1\right)}+\tau_{\mathrm{d\alpha},\sigma }^{\left(2\right)}\right)$, where $\left(\tau_{\mathrm{dL},\sigma }^{\left(1\right)}={\tilde{g}}_{\mathrm{d\sigma}}^{r}V_{R}^{*}g_{R}^{r}We^{-\mathrm{i\phi}}g_{L}^{r}\right)$ and $\left(\tau_{\mathrm{dL},\sigma }^{\left(2\right)}={\tilde{g}}_{\mathrm{d\sigma}}^{r}V_{L}^{*}g_{L}^{r}\right)$ with ${\tilde{g}}_{\mathrm{d\sigma}}^{r}=\frac{1}{1+\chi^{2}}{\left[\omega -\tilde{\epsilon }+i({\tilde{\Gamma }}^{L}+{\tilde{\Gamma }}^{R})\right]}^{-1}$. It is evident that the phase difference between the two paths influences the magnitude of $\left(|G_{\mathrm{d\alpha},\sigma }^{r}{|}^{2}\right)$, hence changing the average electron occupation number in the QD. Via a simple calculation, the phase difference can be obtained, i.e., *Δθ*_*dL*,*σ*_=[*θ*_*R*_−*ϕ*] with *θ*_*α*_ being the argument of $\left(g_{\alpha }^{r}\right)$. Similarly, the two transmission paths between lead-*R* and the QD can be given by $\left(\tau_{\mathrm{dR},\sigma }^{\left(1\right)}={\tilde{g}}_{\mathrm{d\sigma}}^{r}V_{L}^{*}g_{L}^{r}We^{\mathrm{i\phi}}g_{R}^{r}\right)$ and $\left(\tau_{\mathrm{dR},\sigma }^{\left(2\right)}={\tilde{g}}_{\mathrm{d\sigma}}^{r}V_{R}^{*}g_{R}^{r}\right)$ with *Δθ*_*dR*,*σ*_=[*θ*_*L*_ + *ϕ*]. So, in the presence of finite magnetic flux, the amplitude of $\left(|G_{\mathrm{dL},\sigma }^{r}{|}^{2}\right)$ is different from that of $\left(|G_{\mathrm{dR},\sigma }^{r}{|}^{2}\right)$. This leads to the different couplings between the QD and the leads. In the extreme case of *ΠρW*=1, the magnitudes of $\left(\tau_{\mathrm{d\alpha},\sigma }^{\left(1\right)}\right)$ and $\left(\tau_{\mathrm{d\alpha},\sigma }^{\left(2\right)}\right)$ are the same. Then, when $\phi =\frac{\Pi }{2}$, the destructive quantum interference between $\left(\tau_{\mathrm{dL},\sigma }^{\left(1\right)}\right)$ and $\left(\tau_{\mathrm{dL},\sigma }^{\left(2\right)}\right)$ causes $\left(|G_{\mathrm{dL},\sigma }^{r}{|}^{2}\right)$ to be equal to zero, which leads to the decoupling of the QD from lead-*L*. However, the quantum interference between $\left(\tau_{\mathrm{dR},\sigma }^{\left(1\right)}\right)$ and $\left(\tau_{\mathrm{dR},\sigma }^{\left(2\right)}\right)$ is constructive since *Δθ*_*dR*,*σ*_=0 in such a case. So, the QD only feels lead-*R* with 〈*n*_*σ*_〉=*f*_*Rσ*_(*ϵ*). Oppositely, for the case of $\phi =\frac{3\Pi }{2}$, only the property of lead-*L* influences the electron in the QD. So far, we have noted that the AB-Fano effect modulates the quantum interference that contributes to the electron distribution in the QDs.

In the following, we incorporate the electron interaction into the calculation. In the case of weak QD-lead coupling, 〈*n*_*σ*_〉 can be expressed in an analytical way, i.e.,

(6) $$\begin{array}{ll} \langle n_{\sigma }\rangle \;=\;\frac{F_{\sigma }(\tilde{\epsilon })\;-\;F_{\bar{\sigma }}(\tilde{\epsilon })F_{\sigma }(\tilde{\epsilon })\;+\;F_{\sigma }(\tilde{\epsilon }\;+\;U)F_{\bar{\sigma }}(\tilde{\epsilon })}{1\;-\;F_{\bar{\sigma }}(\tilde{\epsilon })F_{\sigma }(\tilde{\epsilon })\;+\;F_{\bar{\sigma }}(\tilde{\epsilon })F_{\sigma }(\tilde{\epsilon }\;+\;U)\;+\;F_{\sigma }(\tilde{\epsilon })F_{\bar{\sigma }}(\tilde{\epsilon }\;+\;U)\;-\;F_{\sigma }(\tilde{\epsilon }\;+\;U)F_{\bar{\sigma }}(\tilde{\epsilon }\;+\;U)}, & \; \end{array}$$

in which $\left(F_{\sigma }(\omega )=\frac{\Delta_{-\phi }\Gamma^{L}f_{\mathrm{L\sigma}}+\Delta_{\phi }\Gamma^{R}f_{\mathrm{R\sigma}}}{\Gamma^{L}+\Gamma^{R}}\right)$. Then,

(7) $$\langle n_{s}\rangle =\frac{F_{↑}(\tilde{\epsilon })-F_{↓}(\tilde{\epsilon })+F_{↑}(\tilde{\epsilon }+U)F_{↓}(\tilde{\epsilon })-F_{↓}(\tilde{\epsilon }+U)F_{↑}(\tilde{\epsilon })}{1-F_{\bar{\sigma }}(\tilde{\epsilon })F_{\sigma }(\tilde{\epsilon })+F_{\bar{\sigma }}(\tilde{\epsilon })F_{\sigma }(\tilde{\epsilon }+U)+F_{\sigma }(\tilde{\epsilon })F_{\bar{\sigma }}(\tilde{\epsilon }+U)-F_{\sigma }(\tilde{\epsilon }+U)F_{\bar{\sigma }}(\tilde{\epsilon }+U)}.$$

**Figure 2:**
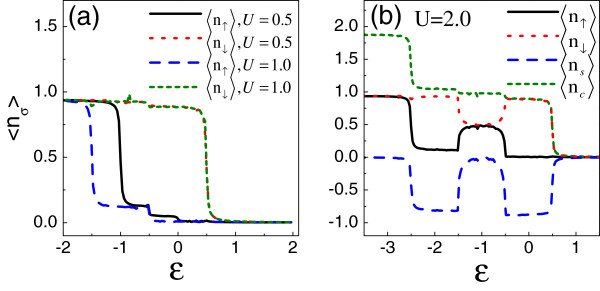
**The influence of Coulomb interaction on the properties of *〈****n*_***σ***_***〉 *(a)** and ***〈****n*_***s***_***〉 *(b)**. The relevant parameters are taken to be *W* = 0.3, *V*_*α*_ = 0.1, and $\phi =\frac{\Pi }{2}$.

In Figure 2, by assuming *W*=0.3 and $\phi =\frac{\Pi }{2}$, we show the spin accumulation in the QD in the cases of $\left(U=0.5\right)$, 1.0, and 2.0, respectively. As shown in Figure 2a, for the cases of *U*≤*eV*_*s*_, the energy region where the spin accumulation emerges is directly widened, and in the whole region of $-\frac{eV_{s}}{2}-U<\epsilon <\frac{eV_{s}}{2}$, the spin polarization is robust. Thus, the intradot Coulomb interaction benefits the spin accumulation in the QD. Such a result can be explained in the following way: When $\phi =\frac{\Pi }{2}$, the QD only ‘couples to’ lead-*R* in which $\mu_{\mathrm{R↓}}=\frac{eV_{s}}{2}$ and $\mu_{\mathrm{R↑}}=-\frac{eV_{s}}{2}$. Consequently, when the QD level *ϵ*is shifted below *μ*_*R↓*_, a spin-down electron will occupy such a level, but at this time, the level *ϵ* + *U* is unoccupied. Next, when the level *ϵ* + *U*is below *μ*_*R↓*_, the Pauli exclusion principle makes it empty, so the spin accumulation in the QD is equal to −1 approximately. Only when the level *ϵ* + *U* decreases to the position of *μ*_*R↑*_does a spin-up electron have an opportunity to occupy it, and then, the spin accumulation disappears. However, with regard to the case of *U*=2.0, we see that with the decrease of *ϵ*to −0.5, the magnitude of spin accumulation goes down deeply, and around the region of *ϵ*=−1.0, the value of 〈*n*_*s*_〉 almost encounters its zero. However, by a further adjustment of *ϵ*_0_to *ϵ*_0_=−1.5, such a spin accumulation then gets close to 1 again. We can understand this phenomenon as follows. Since the strong Coulomb interaction, the levels *ϵ*and *ϵ* + *U* will not be located in the spin bias window simultaneously. Then, they respectively contribute to the spin accumulation. So, in the regions of −0.5<*ϵ*<0.5 and −0.5<*ϵ* + *U*<0.5, there emerges an apparent spin accumulation. However, when the level is shifted around the point of *ϵ*=−1.0, the level *ϵ* + *U* is above *μ*_*R↓*_, so it is unoccupied. Then, the level *ϵ*, which is below *μ*_*R↑*_, will confine the different-spin electrons with the same ability. So far, we have known the role of electron interaction in adjusting the spin accumulation in such a structure.

### The double-QD AB interferometer

**Figure 3:**
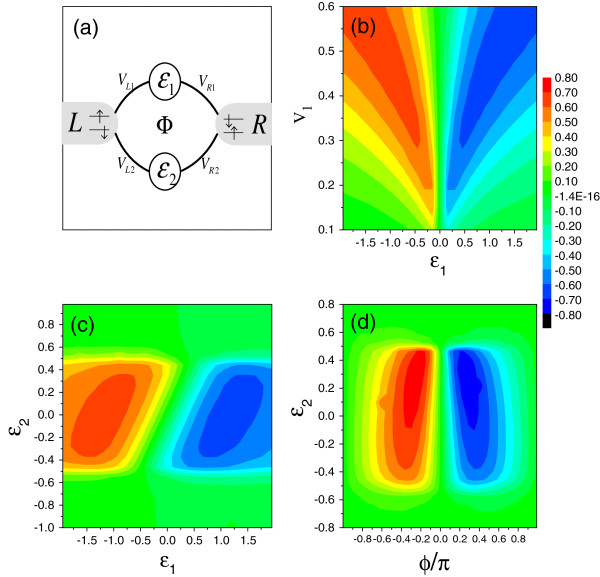
**The AB interferometer with two QDs. (a)** Schematic of an AB interferometer with one QD in each of its arm. **(b)** The spin accumulation in QD-2 influenced by the properties of the other arm. **(c)** The influence of the QD levels on the spin accumulation in QD-2. **(d)** The spin accumulation in QD-2 affected by the local magnetic flux. The spin bias is fixed with *eV*_*s*_ = 1.0.

The AB interferometer with one QD in each of its arm (see Figure 3a) is another typical structure in studying the electron transport behaviors modified by the AB phase. For such a structure, both *H*_*D*_ and *H*_*T*_ have alternative forms as $\left(H_{D}=\underset{\mathrm{j\sigma}}{\sum }\epsilon_{j}d_{\mathrm{j\sigma}}^{‡}d_{\mathrm{j\sigma}}+U_{j}n_{\mathrm{j↑}}n_{\mathrm{j↓}}\right)$, and $\left(H_{T}=\underset{\mathrm{\alpha kj},\sigma }{\sum }V_{\mathrm{\alpha j}}c_{\mathrm{\alpha k\sigma}}^{‡}d_{\mathrm{j\sigma}}+\text{h.c.}\right)$. $\left(d_{\mathrm{j\sigma}}^{‡}\;(d_{\mathrm{j\sigma}})\right)$ is the creation (annihilation) operator of electron in QD-*j*. *ϵ*_*j*_ is the corresponding QD level, and *U*_*j*_ denotes the intradot electron interaction strength. *V*_*αj*_ represents the coupling between QD-*j* and lead-*α*.

Here, we would like to know whether the Fano interference manner is also necessary to achieve the spin accumulation of such a structure. If so, how do the properties of nonresonant channel affect the spin accumulation? Based on such an idea, we begin to analyze the average electron occupation number of QD-*j* by the formula $\left(\langle n_{\mathrm{j\sigma}}\rangle =-\frac{i}{2\Pi }\int \mathrm{d\omega}\overset{<}{\underset{\mathrm{jj},\sigma }{G}}(\omega )\right)$. The lesser Green function *G*^<^(*ω*) also obeys the relationship in Equation 3, and the retarded Green function *G*^*r*^ can be expressed as

(8) $${\left[G_{\sigma }^{r}\right]}^{-1}=\left[\begin{array}{cccc} \;g_{L}^{r-1} & 0 & -V_{L1} & -V_{L2}\\ 0 & g_{R}^{r-1} & -V_{R1} & -V_{R2}\\ -V_{L1}^{*} & -V_{R1}^{*} & \;g_{1\sigma }^{r-1} & 0\\ -V_{L2}^{*} & -V_{R2}^{*} & 0 & g_{2\sigma }^{r-1} \end{array}\right]\;,$$

where $g_{\mathrm{j\sigma}}^{r}=\frac{1-\langle n_{j\bar{\sigma }}\rangle }{\omega -\epsilon_{j}+i0^{+}}+\frac{\langle n_{j\bar{\sigma }}\rangle }{\omega -\epsilon_{j}-U_{j}+i0^{+}}$ within the Hubbard approximation. Surely, Equation 5 is still suitable for evaluating the electron properties of this system.

Without loss of generality, we take QD-2 as an example to investigate the spin accumulation behaviors of such a structure. In the presence of magnetic flux, the coupling coefficients take the following form: *V*_*L*1_=*V*_1_*e*^*iϕ*/4^, $\left(V_{R1}^{*}=V_{1}e^{\mathrm{i\phi}/4}\right)$, *V*_*R*2_=*V*_2_*e*^*iϕ*/4^, and $\left(V_{L2}^{*}=V_{2}e^{\mathrm{i\phi}/4}\right)$. *V*_*j*_ is the strength of the QD-lead coupling. The numerical results are shown in Figure 3b,c,d. In Figure 3b, by fixing $\phi =\frac{\Pi }{2}$, *ϵ*_2_=0, and *V*_2_=0.1, we plot the spectrum of spin accumulation in QD-2 vs *ϵ*_1_and *V*_1_. It is obvious that the increase of *V*_1_can efficiently enhance the spin accumulation in QD-2. This means that in the case of finite spin bias and magnetic flux, a nonresonant channel is necessary to realize the spin accumulation of such a structure. So, it is the AB-Fano effect, but not the AB effect, that promotes the spin accumulation. Besides, it shows that the level of QD-1 plays a nontrivial role in affecting the spin accumulation. To be precise, when the level of QD-1 is shifted around the zero energy point, there will be no spin accumulation in QD-2. When the level of QD-1 departs from the zero energy, finite spin accumulation emerges with its maximum approximately at the position where $V_{1}=0.45\sqrt{\left|\epsilon_{1}\right|}$. The other important result is that the sign of 〈*n*_2*s*_〉 will change when the level *ϵ*_1_exceeds the zero energy point. Therefore, in comparison with the one-QD AB interferometer, we can find that the spin accumulation of this structure can be manipulated flexibly.

Next, we choose *V*_1_=0.6 and investigate the spin accumulation in QD-2 influenced by the change of QD levels, as shown in Figure 3c. We find that similar to the former structure, the spin accumulation occurs only when the corresponding QD level is located in the spin bias window. However, the characteristic of 〈*n*_2*s*_〉 lies where its sign ( + /−) is differentiated by the line of *ϵ*_1_=*ϵ*_2_, where the spin accumulation disappears. This result indicates that if the spin bias is large enough, at the point of *ϵ*_1_=0, the sign of 〈*n*_2*s*_〉 can be altered by the change of *ϵ*_2_. On the other hand, in Figure 3d, we investigate 〈*n*_2*s*_〉 as functions of *ϕ* and *ϵ*_2_. The QD-lead couplings are taken to *V*_1_=5*V*_2_=0.5, and the level of QD-1 is fixed at *ϵ*_1_=1. It is seen that the reversal of the magnetic flux direction can change the sign of spin accumulation, but in such a structure, the level of QD-2 tends to affect the maximum of spin accumulation, which appears around the points of *ϵ*_2_=0.25 and *ϕ*=±0.3*Π*. Thereby, we notice that the properties of the resonant channel, e.g., the level of QD-2, are also important factors to change the magnitude of the spin accumulation.

For such a structure, it is difficult for us to write out the analytical expression of 〈*n*_2*s*_〉. So, we can only present a qualitative discussion to explain the above results by analyzing the quantum interference that contributes to the spin accumulation. Obviously, the expression of $\left(G_{2\alpha ,\sigma }^{r}\right)$ can be written as the summation of two Feynman paths, i.e., $\left(G_{2\alpha ,\sigma }^{r}=\tau_{2\alpha ,\sigma }^{\left(1\right)}+\tau_{2\alpha ,\sigma }^{\left(2\right)}\right)$. Then, the quantum interference feature determines the coupling strength between QD-2 and the leads. However, it is found that

(9) $$\begin{array}{lll} \tau_{2L,\sigma }^{\left(1\right)} & =\overset{\infty }{\underset{j=1}{\sum }}i{\left(-{\tilde{g}}_{2\sigma }^{r}\Gamma_{21}{\tilde{g}}_{1\sigma }^{r}\right)}^{j}\Gamma_{12}^{j-1}{\tilde{V}}_{1L},\; & \;\\ \tau_{2L,\sigma }^{\left(2\right)} & =\overset{\infty }{\underset{j=0}{\sum }}{\tilde{g}}_{2\sigma }^{r}{\left(-{\tilde{g}}_{2\sigma }^{r}{\tilde{g}}_{1\sigma }^{r}\Gamma_{12}\Gamma_{21}\right)}^{j}{\tilde{V}}_{2L},\; \end{array}$$

where ${\tilde{V}}_{\mathrm{j\alpha}}={\tilde{V}}_{\mathrm{\alpha j}}^{*}=V_{\mathrm{\alpha j}}^{*}\sqrt{\Pi \rho }$ and $\left({\tilde{g}}_{\mathrm{j\sigma}}^{r}={\left[\omega -\epsilon_{j}+i\Gamma_{\mathrm{jj}}\right]}^{-1}\right)$ with $\left(\Gamma_{\mathrm{jl}}=\underset{\alpha }{\sum }\Pi V_{\mathrm{\alpha j}}V_{\mathrm{\alpha l}}^{*}\rho \right)$. So, the coupling between QD-2 and lead-*L* is determined by the quantum interference among infinite-order Feynman paths, different from that in the one-QD structure. This inevitably leads to the complicated features of the spin accumulation. Similarly, the three transmission paths between lead-*R* and QD-2 can be given by

(10) $$\begin{array}{lll} \tau_{2R,\sigma }^{\left(1\right)} & =\overset{\infty }{\underset{j=1}{\sum }}i{\left(-{\tilde{g}}_{2\sigma }^{r}\Gamma_{21}{\tilde{g}}_{1\sigma }^{r}\right)}^{j}\Gamma_{12}^{j-1}{\tilde{V}}_{1R},\; & \;\\ \tau_{1R,\sigma }^{\left(2\right)} & =\overset{\infty }{\underset{j=0}{\sum }}{\tilde{g}}_{2\sigma }^{r}{\left(-{\tilde{g}}_{2\sigma }^{r}{\tilde{g}}_{1\sigma }^{r}\Gamma_{12}\Gamma_{21}\right)}^{j}{\tilde{V}}_{2R}.\; \end{array}$$

Despite the complicated quantum interferences among infinite paths, we try to clarify the quantum interference feature by calculating the phase differences between the lowest-order paths. This is because the quantum interference among lowest-order paths contributes mainly to the coupling between QD-2 and the leads. For instance, the three lowest-order paths between QD-2 and lead-*L* are $\left(\tau_{2L,\sigma }^{\left(1,a\right)}=-i{\tilde{g}}_{2\sigma }^{r}{\tilde{V}}_{2L}{\tilde{V}}_{L1}{\tilde{g}}_{1\sigma }^{r}{\tilde{V}}_{1L}\right)$, $\left(\tau_{2L,\sigma }^{\left(1,b\right)}=-i{\tilde{g}}_{2\sigma }^{r}{\tilde{V}}_{2R}{\tilde{V}}_{R1}{\tilde{g}}_{1\sigma }^{r}{\tilde{V}}_{1L}\right)$, and $\left(\tau_{2L,\sigma }^{\left(2,0\right)}={\tilde{g}}_{2\sigma }^{r}{\tilde{V}}_{2L}\right)$, and the phase differences are $\left(\Delta \theta_{2L,\sigma }^{\left(a,b\right)}=\phi \right)$, $\Delta \theta_{2L,\sigma }^{\left(a,0\right)}=\theta_{1}+\frac{\phi }{2}-\frac{\Pi }{2}$, and $\Delta \theta_{2L,\sigma }^{\left(b,0\right)}=\theta_{1}-\frac{\Pi }{2}$, respectively, with *θ*_*j*_ being the argument of $\left({\tilde{g}}_{\mathrm{j\sigma}}^{r}\right)$. By a same token, we have the results that $\left(\tau_{2R,\sigma }^{\left(1,a\right)}=-i{\tilde{g}}_{2\sigma }^{r}{\tilde{V}}_{2L}{\tilde{V}}_{L1}{\tilde{g}}_{1\sigma }^{r}{\tilde{V}}_{1R}\right)$, $\left(\tau_{2R,\sigma }^{\left(1,b\right)}=-i{\tilde{g}}_{2\sigma }^{r}{\tilde{V}}_{2R}{\tilde{V}}_{R1}{\tilde{g}}_{1\sigma }^{r}{\tilde{V}}_{1R}\right)$, and $\left(\tau_{2R,\sigma }^{\left(2,0\right)}={\tilde{g}}_{2\sigma }^{r}{\tilde{V}}_{2R}\right)$; and $\left(\Delta \theta_{2R,\sigma }^{\left(a,b\right)}=\phi \right)$, $\Delta \theta_{2R,\sigma }^{\left(a,0\right)}=\theta_{1}+\frac{\phi }{2}+\frac{\Pi }{2}$, and $\left(\Delta \theta_{2R,\sigma }^{\left(b,0\right)}=\theta_{1}+\frac{\Pi }{2}\right)$. For a typical case of *ω*=0, *ϵ*_1_=1, and $\phi =\frac{\Pi }{2}$, we get the result that $\Delta \theta_{2L,\sigma }^{\left(a,b\right)}=\frac{\Pi }{2}$, $\left(\Delta \theta_{2L,\sigma }^{\left(a,0\right)}=\Pi \right)$, and $\Delta \theta_{2L,\sigma }^{\left(b,0\right)}=\frac{3\Pi }{4}$. So, the destructive quantum interference among these paths leads to the decoupling of QD-2 from lead-*L*. In such a case, however, the quantum interference among $\left(\tau_{2R,\sigma }^{\left(1,a\right)}\right)$, $\left(\tau_{2R,\sigma }^{\left(2,b\right)}\right)$, and $\left(\tau_{2R,\sigma }^{\left(0\right)}\right)$ is constructive since $\Delta \theta_{2R,\sigma }^{\left(a,b\right)}=\frac{\Pi }{2}$, $\left(\Delta \theta_{2R,\sigma }^{\left(a,0\right)}=0\right)$, and $\Delta \theta_{2R,\sigma }^{\left(b,0\right)}=\frac{\Pi }{4}$. Thus, the spin bias of lead-*R* determines the spin accumulation in QD-2. Surely, *θ*_1_ is dependent on *ω*, but one should understand that the quantum interference of *ω*=0 makes the main contribution to the spin accumulation. So, the accumulation of this structure. Meanwhile, note that only when the arm of QD-1 offers a nonresonant channel are the magnitudes of the paths close to one another, so that the quantum interference effect is clear.

Next, we demonstrate the effect of *ϵ*_2_on the value of 〈*n*_2*s*_〉. In Equations 9 to 10, one can find that in the higher-order paths, the two arms of the interferometer are visited repeatedly. Then, the properties of the two arms play an important role in affecting the quantum interference. In the study by Gong et al. [63], our calculations showed that when the levels of the two QDs are the same, the quantum interference between the two arms become weak, but only the nonresonant one determines the electron properties of this structure. As a consequence, in such a case, the interferometer can be considered as a single-channel structure, and then, the picture of quantum interference disappears. With this viewpoint, we understand the vanishment of the spin accumulation in the case of *ϵ*_1_=*ϵ*_2_.

**Figure 4:**
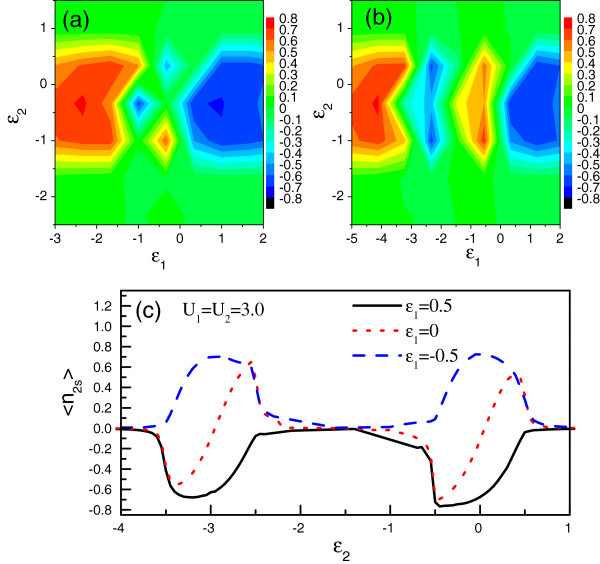
**The influence of Coulomb interactions on the spectra of *〈****n*_***2****s*_***〉***. The relevant parameters are taken to be *V*_1_ = 0.5, *V*_2_ = 0.1, and $\phi =\frac{\Pi }{2}$. **(a)** The spectrum of 〈*n*_2*s*_〉 vs *ϵ*_1_ and *ϵ*_2_. The Coulomb interactions are taken to be *U*_1_ = *U*_2_ = 1.0. **(b)** The spectrum of 〈*n*_2*s*_〉 with *U*_1_ = 3.0 and *U*_2_ = 1.0. **(c)** The curve of 〈*n*_2*s*_〉 vs *ϵ*_2_ with *U*_1_ = *U*_2_ = 3.0. The level of QD-1 is taken to be *ϵ*_1_ = 0 and ±0.5, respectively.

In Figure 4, by choosing *V*_1_=0.5,*V*_2_=0.1, and $\phi =\frac{\Pi }{2}$, we investigate the influence of the intradot Coulomb interactions on the spin accumulation in QD-2. From Figure 4a,b,c, we clearly find that the many-body effect in QD-2 (i.e., the resonant-channel QD) on the spin accumulation is similar to that in the single-QD AB interferometer. Namely, in the case of *U*_2_≤*eV*_*s*_, e.g., *U*_2_=1.0, the energy region where the spin accumulation emerges is directly widened. As a result, the spin polarization is always robust in the whole region of $-\frac{eV_{s}}{2}-U_{2}<\epsilon <\frac{eV_{s}}{2}$. In the case of strong Coulomb interaction, e.g., *U*_2_=3.0 in Figure 4c, the spectrum of 〈*n*_2*s*_〉 vs *ϵ*_2_ is divided into two groups, which are analogous to each other. This result is easy to understand in terms of the analysis about the many-body effect in the above subsection. Alternatively, in Figure 4a,b,c we see that the Coulomb interaction with the nonresonant channel plays a more significant role in modifying the spin accumulation. First, a nonzero *U*_1_ causes the energy region where the positive spin accumulation appears to shift to the low-energy direction, and only when varying *ϵ*_1_to *ϵ*_1_ + *U*_1_≤0 can one see the positive spin accumulation. Secondly, with the further increase of *U*_1_, in the middle-energy region where *ϵ*_1_<0<*ϵ*_1_ + *U*_1_, finite spin accumulation in QD-2 is also observed. For instance, for the case of *U*_1_=3.0, positive 〈*n*_2*s*_〉 comes up around the point of *ϵ*_1_=−0.5, whereas the negative 〈*n*_2*s*_〉 occurs in the vicinity of *ϵ*_1_=−2.5. We explain this result as follows. A finite *U*_1_will lead to *ϵ*_1_splitting into *ϵ*_1_and *ϵ*_1_ + *U*_1_. Accordingly, two nonresonant channels contribute to the quantum interference. When *U*_1_=1.0, the two nonresonant channels have the opportunity to simultaneously act on the quantum interference. In the case of −1.0<*ϵ*_1_<0.0, the sign of *ϵ*_1_ + *U*_1_ is greater than zero. Then, the electron waves in the two channels are phase-opposite, which significantly weakens the quantum interference and suppresses the spin accumulation in QD-2. However, for a strong Coulomb interaction in QD-1, when *ϵ*_1_ is tuned below the zero energy point, the level *ϵ*_1_ + *U*_1_is still much greater than zero. Then in such a case, the Coulomb-induced level contributes little to the quantum interference, and a single-electron interference picture remains. Due to this reason, we find the positive spin accumulation in QD-2 when *ϵ*_1_=−0.5 in Figure 4b,c. On the contrary, when *ϵ*_1_=−2.5, *ϵ*_1_ + *U*_1_=0.5. Then, the coupling between *ϵ*_1_ + *U*_1_ and the leads provides a channel for the quantum interference, leading to the appearance of negative spin accumulation in QD-2. The further decrease of *ϵ*_1_ will cause *ϵ*_1_ + *U*_1_ to be less than zero. Compared with the result of *ϵ*_1_=−2.5, the change of the sign of *ϵ*_1_ + *U*_1_brings about the positive 〈*n*_2*s*_〉. In addition, note that when $\epsilon_{1}=-\frac{U_{1}}{2}$, one can obtain the result of *ϵ*_1_=−*ϵ*_1_ + *U*_1_. Then, the opposite-phase electron waves in the two nonresonant channels contribute zero to the quantum interference, so in such a case, no spin accumulation occurs in QD-2.

## Summary

In summary, we have studied the spin accumulation characteristics of two AB interferometers with QDs embedded in their arms by considering spin bias in the leads. It has been found that regardless of the configurations of the interferometers, the spin accumulations are strongly dependent on the quantum interference features of the interferometers. Namely, the nonresonant transmission ability between the leads and the local magnetic flux can efficiently adjust the spin accumulation properties of the QD. By analyzing the quantum interferences among the Feynman paths, it was seen that the quantum interferences can cause the QD in the resonant channel to be decoupled from one of the leads. Accordingly, the spin bias in one lead will drive the spin accumulation in such a QD. So, it is certain that the AB-Fano effect assists to manipulate the spin accumulation. Further analysis showed that the double-QD interferometer has advantages in manipulating the spin states in the resonant channel. In view of the obtained results, we propose the AB interferometers with QDs to be alternative candidates for spin manipulation in QD devices.

## Competing interests

The authors declare that they have no competing interests.

## Authors’ contributions

WJG designed the theoretical model, deduced the relevant formula, and drafted the manuscript. YH carried out the numerical calculations. GZW participated in the analysis about the results. AD improved the manuscript. All authors read and approved the final manuscript.
